# Technology-Enhanced Learning in the Education of Oncology Medical Professionals: A Systematic Literature Review

**DOI:** 10.1007/s13187-023-02329-1

**Published:** 2023-06-26

**Authors:** Taibe Kulaksız, Jana Steinbacher, Marco Kalz

**Affiliations:** Institute for Arts, Music and Media, Heidelberg University of Education, Keplerstraße 87, 69120 Heidelberg, Germany

**Keywords:** Oncology education, Cancer education, Cancer care professionals, Technology-enhanced learning, Digital education, Systematic review

## Abstract

As cancer continues to be a significant global health challenge, the education of oncology professionals plays a crucial role in providing quality cancer care and achieving optimal patient outcomes. In order to meet the growing need for flexible, accessible, and effective training, this study examines the role of technology-enhanced learning (TEL) in the education of oncology medical professionals. Following the PRISMA guidelines, this systematic review included 34 articles published between 2012 and 2022 in EBSCO and PubMed databases. Findings reveal a diverse range of digital tools being used in oncology training, despite a shortage of advanced educational technologies and limited functional improvement compared to traditional instruction. Since the training primarily targeted at multiple professions in the medical expert role, with radiation oncologists being overrepresented, other oncology domains should be examined more thoroughly in the future, taking into account distinct professional abilities, e.g. communication, collaboration, and leadership skills with reference to the CanMEDS framework. Although the training programmes generally resulted in positive outcomes according to the Kirkpatrick evaluation model, experimental research designs were rather limited. Therefore, the substantial contribution and limitations of TEL in oncology education need to be clarified. Precise reporting of digital tools and instructional processes, as well as challenges encountered, is highly recommended to increase transparency and replicability. Research methodology in digital oncology education remains a major concern and should be addressed accordingly in future research.

**Supplementary Information:**

The online version contains supplementary material available at 10.1007/s13187-023-02329-1.

## Introduction

Cancer still is a major public health concern worldwide and the second leading cause of premature mortality and morbidity in Europe. With nearly 3 million new cases and more than 1.2 million patients losing their lives in the European Union every year [1], cancer also accounts for more than 30 million disability-adjusted life years (DALYs, i.e. years of healthy life lost) in Europe [2] and entails a total direct healthcare expenditure exceeding €100 billion annually, an amount which has increased by more than 30% over the last three decades [3].

To address this situation, the education of oncology professionals plays a crucial role in providing quality cancer care and achieving optimal patient outcomes across the patient journey. Europe’s Beating Cancer Plan stresses the importance of a high-quality workforce and proposes the launch of an “inter-specialty cancer training programme” (ISCTP) [4]. In the context of the EU-funded project INTERACT, this training programme is currently being developed, and an initial version of a competence framework has been published [5].

Several partners from the technology-enhanced learning (TEL) domain are involved in this European initiative to ensure the most flexible, accessible, and effective delivery of training to multidisciplinary teams of healthcare professionals. In order to understand the current use of TEL in oncology education and its effects on patient care, to identify strengths, weaknesses, and potential gaps, and to contextualise our research, we conducted a systematic literature review.

Earlier reviews either focus on online education for nurses and allied health professionals, providing an overview of existing studies until 2015[6], or on specific skill development such as online communication training[7]. Our systematic review followed a multidisciplinary and multiprofessional cancer management approach, taking into account that modern cancer care involves a wide range of disciplines and professions across the cancer continuum for the successful delivery of cancer prevention, diagnosis, treatment, care, follow-up, and survivorship care (e.g. pathologists, medical oncologists, radiation oncologists, surgical oncologists). Furthermore, we examine TEL from a broader perspective; i.e. our review does not only focus on the delivery of education and training via online platforms, it also includes mHealth applications, augmented reality, or other advanced learning technologies.

Following a mapping/scoping review type [8, 9], our research questions can be outlined as follows:

1. What types of digital tools are used in oncology education?
2. What skills of medical professionals in oncology are targeted with TEL?
3. What is the scope of research methodology in oncology education?
4. What effects does TEL have on teaching/learning processes in oncology education?
5. What are the publication patterns of research on TEL in oncology education?

## Research Method

This systematic review followed the guideline proposed in the Preferred Reporting Items for Systematic reviews and Meta-Analyses (PRISMA) statement [10]. The PRISMA 2020 checklist and flow diagram were applied to select and examine eligible studies.

## Defining Eligibility Criteria

Our review aimed at providing an overview of TEL in the education of oncology professionals. Inclusion and exclusion criteria to select eligible articles are presented below (Table 1).

**Table 1:** Inclusion/exclusion criteria

	Inclusion	Exclusion
Research design	Empirical	Non-empirical
Objective and sample	Education/training of oncology health professionals	Non-oncological Non-oncological health professionals Non-health professionals Non-educational
Instructional setting	Technology-enhanced learning	Non-technological
Publication type	Journal article (peer reviewed)	Other publication types
Publication year	2012–2022 (November)	Out of this range
Language	English	Other languages
Access	Access to full paper	No access to full paper

First, only empirical studies were included regardless of qualitative, quantitative, or mixed designs. Non-empirical studies (e.g. opinion essays) were excluded. Second, as our review focused on the education/training of oncology health professionals (e.g. medical doctors, pathologists, radiologists, radiation therapists, surgeons, nurses working in oncology-related departments), studies including non-oncological health professionals (e.g. pharmacists) or non-health professionals (e.g. patients, cancer survivors, caregivers, community health workers, general public, non-medical students) were not included. In this sense, cancer prevention and cancer awareness programmes were also not considered as eligible. Studies including undergraduate medical students were eligible if the training was directly related to cancer. Each study composed of mixed target groups was discussed by the researchers as to inclusion. Third, if studies utilised or developed digital tools (e.g. software, apps), they needed to have an educational purpose; otherwise, they were not included. Entirely non-technological instructional settings were excluded.

## Data Sources and Search Strategies

EBSCO and PubMed were selected as databases. The following databases were selected within EBSCO: APA PsycInfo, ERIC, Library, Information Science & Technology Abstracts, MEDLINE, PSYNDEX Literature with PSYNDEX Tests, and Teacher Reference. A filter was applied for publication type (peer reviewed articles) and year (2012–2022). The language of the articles was restricted to English only.

The following keywords and operators were implemented as search strategies in both databases in order to reach eligible papers:

- (“oncology education” or “oncology training” or “cancer education” or “cancer training”) AND (“technology” or “digital”)
- (“professional development”) AND (“technology” or “digital”) AND (“cancer” or “oncology”)

**Fig. 1:**
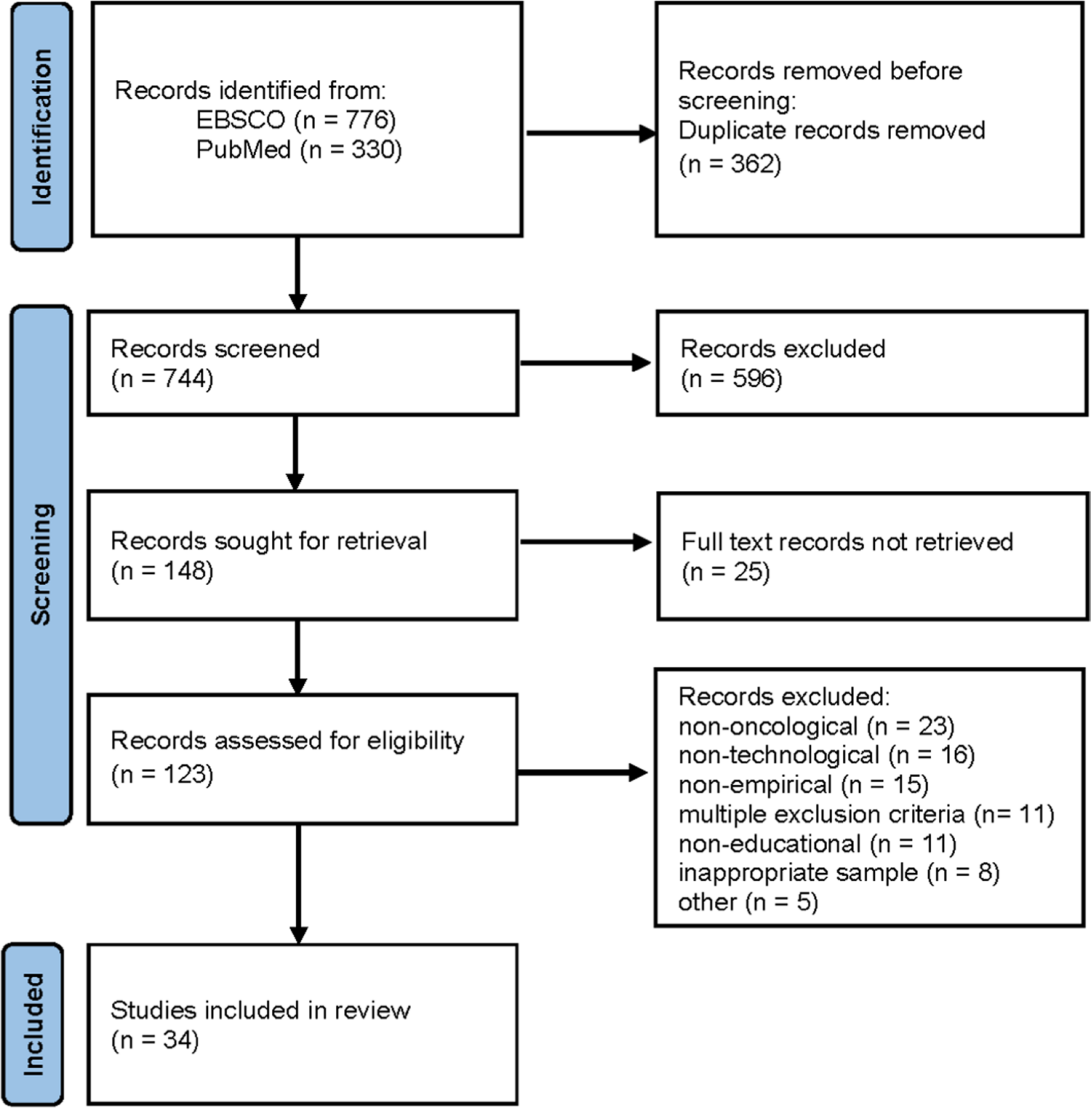
Article selection process based on PRISMA 2020

The selection process of the articles follows the PRISMA 2020 flowchart as shown in Fig. 1. Two researchers worked together during the identification and screening phase to decide on eligibility and inclusion of papers in the review. The search resulted in 1106 records (*N*_EBSCO_ = 776, *N*_PubMed_ = 330). Sixty-four duplicates were removed by EBSCO automatically, i.e. EBSCO records decreased to 712. Search results of both databases were merged and screened manually by the researchers, resulting in additional 298 duplicates. Then, 744 records were examined simultaneously by the researchers based on their titles and abstracts. Five hundred ninety six out of topic articles were excluded. One hundred forty-eight records were sought for retrieval, 25 records could not be retrieved as full text. The remaining articles (*N* = 123) were assessed for eligibility according to the inclusion and exclusion criteria. Articles were removed if the education/training was not cancer care related (*N* = 23), study did not include digital tools for educational purposes (*N* = 16), study was non-empirical (*N* = 15), study was not related to education/training (*N* = 11), the sample was inappropriate (*N* = 8). Eleven articles were excluded due to multiple exclusion criteria. Lastly, 5 articles were excluded due to other reasons (e.g. other systematic reviews, need analyses). As a result, it was decided to include 34 papers in this systematic review.

## Data Collection and Analysis

Following the PRISMA model, data items (outcome domains and other variables) were listed and defined based on the research questions and previous studies. An excel sheet was created as a data collection form. At the beginning, the articles were reviewed by two researchers independently. After the first review, the researchers combined some data items due to missing information. A second examination was conducted based on the revised form (Appendix 1). At the end, all articles were reviewed by the two researchers. A third researcher was consulted when disputes arose or when no consensus could be reached. Finally, all categories were counted to report frequencies and percentages based on the number of studies.

## Findings

Thirty-four articles were included in this systematic review to explore TEL in the education of oncology medical professionals. The following findings provide insights into the (1) type of digital tools used in oncology education, (2) type of knowledge and skills targeted with TEL, (3) scope of the research papers, (4) effects of TEL on teaching/learning processes, and (5) publication patterns of research on TEL in oncology education. A more detailed overview of each article is presented in Appendix 2.

## Digital Tools and Delivery Modes

**Fig. 2:**
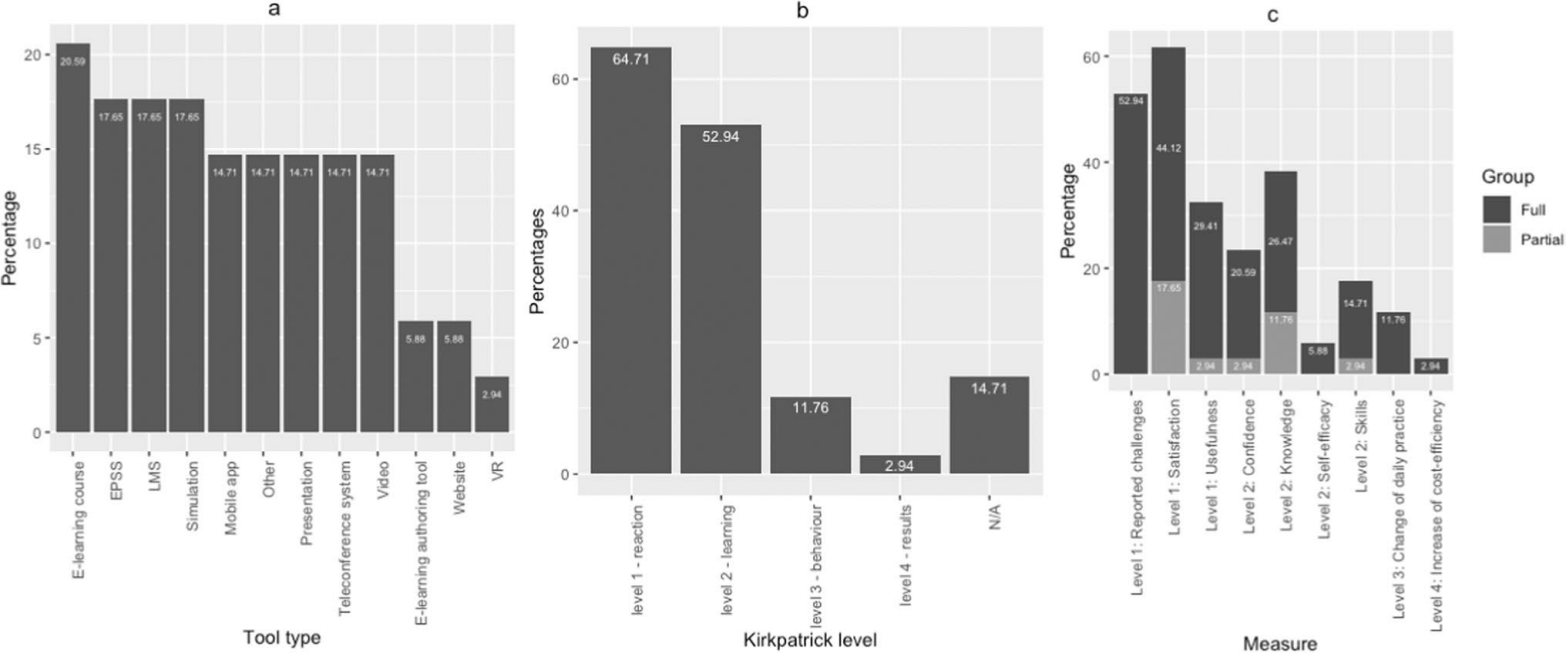
**a** Digital tool type,** b** Kirkpatrick levels, and** c** Kirkpatrick measures

All digital tools used for educational purposes in the studies were categorised by the researchers. These tools mainly referred to e-learning courses, electronic performance support systems (EPSS), learning management systems (LMS), simulations, mobile applications, visual representations (e.g. presentations), and teleconference systems (Fig. 2a). Approximately half of the studies administered more than one digital tool during their training processes (*N* = 16).

23,53% of the studies (*N* = 8) aligned their research purposes and learning objectives with software/application design and development. Among the reviewed articles, the following products were developed: website, simulation, mobile app, e-learning course, chatbot, EPSS, and virtual reality (VR). However, most of the studies used already existing tools for their educational research (76,47%, *N* = 26).

E-learning courses dominated the digital tools in oncology training (20,59%, *N* = 7), e.g. “Oncology Patient Navigator: The Fundamentals”. However, six papers did not specify the courses used. Some e-learning modules were combined with a chatbot (*N* = 1) or quiz (*N* = 1).

The following systems were classified as EPSS (17,65%): BREAST (*N* = 1), supporting cancer detection when reading mammograms, eContour (*N* = 2), used in radiation oncology training, incident learning system (*N* = 1), including multiple professionals of the department to reduce the occurrence of near misses/incidents (e.g. in imaging for planning, planning, and plan transfer), and Varian Eclipse (*N* = 2), used in radiotherapy training. Another six studies (17,65%) utilised LMS to support their training programmes, including ATutor (*N* = 1), Blackboard (*N* = 2), LAMS (*N* = 1), LäraNära (*N* = 1), Moodle (*N* = 2), and Vula (*N* = 1). Two out of these studies used more than one or changed their LMS throughout their educational programmes. Simulations (17,65%) were mainly used for technical skills practice, indicated as Elekta XVI (*N* = 1), PBT TRUS training simulator (*N* = 1), radiation therapy training platform (*N* = 1), VERT (*N* = 2), and virtual hospital (*N* = 1).

M-OncoEd (*N* = 2) and QStream (*N* = 2) were used as mobile applications to support distance education, while TurningPoint (*N* = 1) was used as an add-on to presentations to enhance a blended learning environment. Presentations were mainly delivered via Microsoft PowerPoint (*N* = 4); one paper did not specify the software used. Other examples of visual representations include digital images (*N* = 1), PDF (*N* = 1), videos (*N* = 5), and 360-degree videos (*N* = 1) used as VR. Teleconference systems such as WebEx (*N* = 1) and Zoom (*N* = 2) facilitated blended learning and synchronous parts of distance education [not indicated (*N* = 1)]. There was also an example of an asynchronous discussion board (*N* = 1), hosted via Microsoft Live (*N* = 1).

Two studies (5,88%) utilised e-learning authoring tools, Adobe Captivate (*N* = 1) and Articulate Storyline (*N* = 1), to create digital content. Another two studies (5,88%) included websites, indicated as online toolkit (*N* = 1), forum (*N* = 1), and wiki (*N* = 1) in Appendix 2. Yet, these offered rather limited interaction options.

Most studies did not explicitly name their delivery mode of the training. Therefore, our classifications (distance, blended, or face-to-face) were based on tool development purposes or the description of training processes. 55,88% of the training was classified as distance education, including synchronous and asynchronous modes (*N* = 19). The studies usually used e-learning modules, mobile apps, and LMS as a tool. 29,41% of the training was delivered face-to-face (*N* = 10), using technology to enhance learning and instruction, e.g. EPSS, visual representations, and simulation. Blended learning was employed in 20,59% of the studies (*N* = 7). All of them used more than one educational technology and commonly were composed of LMS or teleconference systems along with other digital tools.

## Educational Context and Sample Characteristics

When analysing the educational context and learning outcomes of the different trainings based on the CanMEDS framework [11], it was observed that the majority of the studies aimed at the “Medical Expert” domain (79,41%, *N* = 27). While some studies focused on specific topics such as cancer pain assessment, cancer screening, or cancer management and treatment, some developed a more general, technology-enhanced oncology curriculum. The “Communicator” role was targeted in 11,76% of the articles (*N* = 4), involving communication and information-provision skills as well as feedback delivery practices. Understanding the value and contribution of different fields within oncology, e.g. with regard to patient navigation, the “Collaborator” role was part of 5,88% of the studies (*N* = 2). Teaching modules relating to the “Professional” role were included in two papers (5,88%). “Leadership” skills were only targeted in one study (2,94%), involving radiation oncologists. The findings pointed out that some articles focused on more than one CanMEDS role [12]. However, we did not come across any research involving the “Health Advocate” or “Scholar” profile among the reviewed studies.

The target groups were classified as professionals, residents, and undergraduate medical students who study oncology related subjects. The training programmes were mainly for professionals (73,53%, *N* = 25), and 29,41% (*N* = 10) were part of oncology residents’ formal/complementary education. A few of the lectures were particularly designed for undergraduates’ formal education or for a broader target group accepting undergraduates as participants (11,76%, *N* = 4), i.e. some studies aimed at more than one educational level [13].

In relation to the different professions within oncology, the samples were distributed as follows: 17,65% radiation oncologists (*N* = 6), 8,82% primary care physicians (*N* = 3), and 8,82% medical students (*N* = 3), colorectal surgeons, exercise specialists, medical physicists, nurses, oncologists, pathologists, and radiologists (2,94%, *N* = 1, respectively). It was observed that usually multiple oncology-related medical departments were recruited for the training rather than one particular target group (44,12%, *N* = 15).

## Research Methodology

We defined the scope of the papers as evaluative, comparative, or developmental. 41,18% of the studies (*N* = 14) aimed at evaluating the effectiveness of TEL and/or digital tools in a single group with pre-/post-intervention assessments. The comparative studies employed experimental research designs to reveal differences between at least two different training approaches (17,65%, *N* = 6). Due to our empirical research approach, there was no article solely focusing on the development of a digital training course and/or tool. 35,29% of the studies (*N* = 12) aimed at both development and evaluation. Initially, digital training courses and/or tools were developed. Then, training courses and/or tools were evaluated to reflect on the efficacy of the outcome (single group interventions). Only one study (2,94%) compared the effectiveness between two different recruitment approaches after developing a digital tool. Lastly, one study (2,94%), examining learner viability and the dynamics of transactions in an online continuing professional development course from an institutionalist view [14], could not be classified.

## Effects of TEL on Teaching/Learning Processes

The findings of the included studies are analysed based on the Kirkpatrick model of training evaluation as presented in Fig. 2b and c, since this model is widely known and applied in the medical domain [15]: reaction (level 1), learning (level 2), behaviour (level 3), and results (level 4).

Participants’ reactions regarding the digital training/tools were examined in 64,71% of the articles (*N* = 22). While participants considered that the training was satisfactory in 44,12% of the articles (*N* = 15), 17,65% of the papers (*N* = 6) reported partially successful training according to the learners’ opinions. Only 11 studies assessed the usefulness of digital tools/software. In 29,41% of the studies (*N* = 10), digital tools were found to be useful as indicated by the learners’ reactions; in 2,94% of the studies (*N* = 1), digital tools were found to be only partially useful. More than half of the papers reported challenges that have been experienced during the implementation of the digital training (52,94%, *N* = 18), e.g. [16].

Training effectiveness was measured in 52,94% of the papers (*N* = 18) in terms of knowledge and skills improvement, confidence, and self-efficacy. While 26,47% and 14,71% of the articles reported that the learners’ knowledge (*N* = 9) and skills (*N* = 5) improved, findings indicated in 11,76% and 2,94% of the studies that knowledge and skills only improved partially (*N* = 4 and *N* = 1, respectively). Confidence levels (*N* = 7) and self-efficacy (*N* = 2) increased in 20,59% and 5,88% of the studies. Confidence increased partially in 2,94% of the papers (*N* = 1).

11,76% of the studies aimed at level 3 (*N* = 4). Those results indicate that digital training changed the learners’ daily professional practice in a positive way. Only one study (2,94%) focused on level 4, in which two different recruitment approaches were compared with regard to cost-efficiency [17]. Five studies were excluded at this point of analysis, either due to lack of data or lack of fit to the Kirkpatrick framework.

## Research Trends

It was generally observed that empirical research in oncology education with TEL is slightly increasing: 2012 (*N* = 3); 2014, 2015, 2016, 2015 (*N* = 1, respectively); 2018 (*N* = 5); 2019 (*N* = 4); 2020 (*N* = 2); 2021 (*N* = 9); and 2022 (*N* = 7). Exploring the countries where the studies were conducted, we counted authors’ affiliations (including cross-national affiliations) to represent the study distribution on a global level. Authors were predominantly represented from the USA (*N* = 62), Australia (*N* = 39), India (*N* = 27), Canada (*N* = 21), and Russia (*N* = 20) (details in Appendix 3). Europe, South America, Africa, and the Middle East have limited research in this field.

The research designs were classified as quantitative, qualitative, or mixed. A more complex methodological classification could not be applied due to an overall lack of description of research designs and underlying paradigms. It was found that mixed methods, combining qualitative and quantitative data, were a prevalent approach among the reviewed articles (*N* = 15). There were 13 studies following quantitative approaches. Qualitative research designs were only implemented in five studies. One study could not be classified.

The majority of the studies used multiple data collection tools (*N* = 18). Surveys were the most important instrument (*N* = 25), sometimes including open-ended questions. Knowledge tests were the second most important tool as to measuring learners’ academic performance, usually carried out as pre-/post-tests. Besides surveys and knowledge tests, data collection methods also included system data logging (*N* = 7), interviews (*N* = 7), focus groups (*N* = 4), expert panels (*N* = 3), observations (*N* = 3), user testing (*N* = 1), and performance tests (*N* = 1). In two studies, data collection remained unclear.

## Discussion and Conclusion

Cancer continues to be one of the most urgent health problems in the world, and the training of health professionals working in oncology is crucial to ensure the most effective care for cancer patients. In this context, the project INTERACT-EUROPE was initiated to provide an inter-specialty cancer training programme with TEL scenarios. This study aimed at examining previous practices of technology-enhanced training in oncology. Following the PRISMA model, 34 empirical articles were investigated in terms of digital tools, delivery modes, learning objectives, sample characteristics, scope of research methodology, effects of TEL on teaching/learning processes, and research trends.

It was found that a variety of digital tools were integrated into oncology education. On the one hand, many studies focused on using generic tools such as teleconference systems, LMS, and e-learning courses for basic knowledge transfer. Most of the studies applied existing technological tools rather than modifying or creating new learning material. Visual digital materials (e.g. presentations, images, videos) generally served as a direct substitute of traditional methods, either with no functional improvement at all (substitution) or with some functional improvement (augmentation) [18]. Therefore, the digital competencies of educators (e.g. digital teaching strategies, selection of learning material, integrating technology into instruction, learning management, assessment) are considered as critical skills for meaningful and interactive digital oncology training [19, 20].

On the other hand, domain-specific mobile apps, EPSS, and simulations were considered to be more progressive in achieving particular skills. However, it was observed that advanced technologies like AR/VR/XR, AI-supported tools, and adaptive technologies were absent in training programmes. There is a clear need for domain-specific and context-oriented educational software. As to design and development of digital tools and applications, the Triple-S framework is highly recommended in terms of scalable, sustainable, and serviceable practices in educational technology [21]. Yet, digitalization in education should focus on pedagogical needs instead of tech-savvy applications.

Though blended learning combines the advantages of distance and face-to-face instruction modes [22], it receives little attention in oncology education. For instance, Morgan and colleagues tested the flipped classroom method to teach gynaecological oncology topics to medical students [23]. Results indicated no significant differences compared to traditional teaching. Initially, it was intended to classify different types and combinations of instruction modes as data items for this systematic review, yet due to ambiguous descriptions, it was not possible to distinguish between different types and combinations of blended approaches, e.g. Staker and Horn’s blended learning taxonomy [22] or Martin and colleagues’ online learning models [24].

Another challenge we faced during the reviewing process was the limited description of digital tools and instructional processes. Even though authors/practitioners usually reported the digital tools, it was not sufficiently described how they were implemented. Therefore, data items regarding elements/features of digital tools and instructional design (e.g. interaction, communication, assignments) needed to be excluded as they were not suitable for further analyses. These findings imply that the replicability of the interventions and the effective use of instructional strategies and digital materials might be threatened. Future research should take into account precise reporting and reflection of advanced digital training contexts.

Among the reviewed articles, we identified many different specialities being involved in the training programmes; almost half of the studies were composed of multiple departments. Yet, radiation oncology-related branches played a prominent role. Technology adaptation in radiation oncology education might be accomplished more effortlessly due to general visualisation needs. Other cancer care divisions such as medical or surgical oncology should be examined more thoroughly in the future. Providing a safe learning environment, especially VR simulations might be beneficial for the procedural skills development of healthcare professionals [25]. Apart from that, the studies usually concentrated on the medical expert domain based on the CanMEDS framework. Other roles of oncology professionals seemed to be neglected. Hence, it is highly recommended to support distinct professional abilities via technology-enhanced learning systems, e.g. communication, collaboration, and leadership skills.

Regarding the methodological scope of the studies, findings reveal that experimental research designs were rather limited. This causes a lack of understanding of the contribution and limitations of TEL in oncology education. Due to the fact that most of the studies employed single group interventions, the effectiveness of digital education/digital tools, comparing traditional and innovative approaches, requires more attention in the future. Thus, experimental studies to control potential biases and to examine the longitudinal impact of online cancer education are needed [6]. Long-term comparative studies are strongly recommended to avoid the “novelty effect” and misleading results of TEL [26]. Furthermore, there is a need for more explorative studies on the pedagogical value of technology integration in oncology education. In that way, the nature of technology-enhanced oncology education can be uncovered not only in terms of technology as digital tools but also in digital teaching/learning methods (e.g. gamification, flipped classroom, online collaborative learning) and digital assessment/evaluation (e.g. e-portfolios, learning analytics). In addition, it might be valuable to take into account course participants characteristics with respect to training preferences in online education, e.g. synchronous and asynchronous learning [27].

The results of the studies were classified according to the Kirkpatrick model of training evaluation. Findings indicate that the training evaluations mainly aimed at level 1 (reaction) and 2 (learning). The learners’ reactions and learning outcomes were overall positive. Data collection tools primarily consisted of self-assessment questionnaires, and results were presented descriptively. However, accredited knowledge tests as well as reliable and validated measurement instruments would contribute to stronger evidence. The absence of reliability and validity studies was also reported in previous systematic reviews [6]. Although learner confidence and self-efficacy are two of the most important outcomes of education, we recommend performance-based and formative evaluations to support their learning rather than self-reported summative evaluations. In addition, challenges during the implementation of educational processes were reported in more than half of the studies, leading to implications for future research in the field of technology-enhanced oncology education. We explicitly support the documentation of challenges besides examples of best practices to stimulate improved pedagogical practice.

Level 3 (behaviour) and 4 (results) studies were generally missing. A limited number of studies reported learners’ behavioural changes [28–31]. As most studies did not pursue long-term interventions or follow-up, it remains unclear which interventions are most effective in generating long-lasting outcomes and potential benefits for patients. Therefore, the actual impact of digital education on professional practice needs to be further investigated. This issue was also raised by Campbell and colleagues, who suggest that participants’ experiences and views, rather than quantitative data and patient-reported outcomes, are weak points in the evidence of clinical and educational effectiveness [6]. Besides, impact studies should also aim at level 4 as it quantifies the institution-wide impact of the training, allowing educators to calculate the return on investment and determine the long-term effectiveness of the programme.

In conclusion, findings of this systematic review demonstrate a diverse range of digital tools being used in the education of oncology medical professionals, despite a shortage of advanced educational technologies. Blended learning was found to be the least utilised delivery mode of instruction. While the training generally resulted in positive outcomes in terms of learning and satisfaction, the self-reported nature of the data demands closer consideration. It is important to recognise that technology in teaching and learning goes beyond access or replacement. Therefore, the pedagogical consequences of technology inclusion in education require educators and learners to develop digital skills, as well as a redistribution of roles and responsibilities in the educational process.

This study has some limitations as it only used two databases and a specific set of search terms, and full-text-access papers, which might have led to the omission of relevant articles. Furthermore, the extracted findings were constrained by the reliability and validity of the examined articles. Although we initially aimed to include more data items such as research design, variables, learning outcomes, and data analysis, we were unable to strictly adhere to the PRISMA guidelines and decided to merge some data items. Research methodology in digital oncology education remains a major concern and should be addressed accordingly in future research.

### Supplementary Information

Below is the link to the electronic supplementary material.

Supplementary file1 (DOCX 20 KB)

Supplementary file2 (XLSX 90 KB)

Supplementary file3 (DOCX 132 KB)

## Data Availability

Data is available within the article or its supplementary material.
