# Supplementary material for: Technology-Enhanced Learning in the Education of Oncology Medical Professionals: A Systematic Literature Review
Source: J Cancer Educ. 2023 Jun 26;38(5):1743–51. doi: 10.1007/s13187-023-02329-1 (PMC10509048; doi:10.1007/s13187-023-02329-1)
Supplement: Supplementary file 1 — Supplementary file1 (DOCX 20 KB) [file 13187_2023_2329_MOESM1_ESM.docx]

| **Data Items** | **Description** | **Synthesis method explanations** |
| --- | --- | --- |
| Year | The year the study was published and included in an issue. | If the study is not included in an issue, the online publication year was reported. |
| Journal | Full name of the journal where the article was published. | - |
| Title | Full name of the published article. | - |
| Country | Countries represented by the authors in the article. | If authors are affiliated with more than one country, all of them were reported. |
| Research purpose | Classification as developmental, evaluative, or comparative based on research aim and research questions/hypothesis. | Developmental: These studies aim at the design/development of digital tools/material or digital training.  Evaluative: These studies aim to test the effectiveness of TEL and/or digital tools in a single group, usually with pre-/post-intervention assessments.  Comparative: These studies aim to compare the effectiveness of TEL and/or digital tools between at least two different training approaches, usually carried out as experimental research designs with control groups. |
| CanMEDS roles | Classification of the learning outcomes targeted in the educational program according to the CanMEDS framework. | Reported as Medical Expert, Communicator, Collaborator, Leader, Health Advocate, Scholar, Professional  Classification was based on research aim, variables, and targeted learning outcomes |
| Digital tool development | Development of a new digital tool/software for teaching/learning purposes. | Reported as yes / no  Usage of existing digital tools/software was reported as no |
| Digital tool type | All digital tools used in the study for teaching/learning purposes. | E-learning authoring tool: e.g. Adobe Captivate  E-learning courses  E-Performance Support System (EPSS): e.g. eContour, Varian Eclipse  Learning Management System (LMS): e.g. Blackboard, Moodle  Mobile app: e.g. M-OncoEd, Qstream  Simulation: e.g. VERT, Virtual Hospital  Teleconference system: e.g. WebEx, Zoom  Virtual Reality (VR): e.g. 360-degree videos  Visual representation: e.g. presentations, digital images, videos  Website: e.g. wiki, forum, toolkit |
| Delivery mode | The mode of delivery of instruction. | Reported as distance / blended / face-to-face |
| Research design | The research methodology followed in the study. | Reported as quantitative / qualitative / mixed  Decision based on data collection tools, unless it is specified in the study |
| Level of education | The seniority of the target group. | Reported as undergraduate / resident / professional |
| Sample (profession) | The professional department of the target group. | The profession was directly reported, if the sample consisted of a single profession. Otherwise, it was reported as “multiple”. |
| Data collection tools | All data collection instruments used in the study. | Classified as survey, knowledge test, performance test, interview, focus group, expert panel, observation, system data log, user testing |
| Kirkpatrick model of training evaluation | Classification of results of the training based on the Kirkpatrick model of training evaluation. | Level 1- Reaction: Participants’ level of satisfaction with the digital training/tool.  Level 2- Learning: Improvement of knowledge, skills, confidence, or self-efficacy.  Level 3- Behaviour: Transfer of acquired skills into professional practice.  Level 4- Results: Impact of training on an institutional level.  N/A: There were no solid findings presented / results were not applicable for the Kirkpatrick model of training evaluation. |
