# Supplementary material for: Technology-Enhanced Learning in the Education of Oncology Medical Professionals: A Systematic Literature Review
Source: J Cancer Educ. 2023 Jun 26;38(5):1743–51. doi: 10.1007/s13187-023-02329-1 (PMC10509048; doi:10.1007/s13187-023-02329-1)

**Table 1: Digital tools and delivery modes (N=34)**

|  | **f** | **%** |
| --- | --- | --- |
| **Digital tool development** | | |
| Yes | 8 | 23,53 % |
| No | 26 | 76,47 % |
| **Digital tool type** | | |
| E-learning authoring tool | 2 | 5,88 % |
| E-learning course | 7 | 20,59 % |
| EPSS | 6 | 17,65 % |
| LMS | 6 | 17,65 % |
| Mobile app | 5 | 14,71 % |
| Presentation | 5 | 14,71 % |
| Simulation | 6 | 17,65 % |
| Teleconference system | 5 | 14,71 % |
| VR | 1 | 2,94 % |
| Website | 2 | 5,88 % |
| Video | 5 | 14,71% |
| Other | 5 | 14,71% |
| **Instruction delivery mode** | | |
| Distance | 19 | 55,88 % |
| Blended | 7 | 20,59 % |
| Face-to-face | 10 | 29,41 % |

**Table 2: Educational context & sample characteristics**

|  | **f** | **%** |
| --- | --- | --- |
| **CanMEDS roles** | | |
| Medical Expert | 27 | 79,41 % |
| Communicator | 4 | 11,76 % |
| Collaborator | 2 | 5,88 % |
| Leader | 1 | 2,94 % |
| Health Advocate | - | - |
| Scholar | - | - |
| Professional | 2 | 5,88 % |
| **Level of education** | | |
| Professionals | 25 | 73,53 % |
| Residents | 10 | 29,41 % |
| Undergraduates | 4 | 11,76 % |
| **Sample (profession)** | | |
| Colorectal surgeons | 1 | 2,94 % |
| Exercise professionals | 1 | 2,94 % |
| Medical physicists | 1 | 2,94 % |
| Medical students | 3 | 8,82 % |
| Nurses | 1 | 2,94 % |
| Oncologists | 1 | 2,94 % |
| Pathologists | 1 | 2,94 % |
| Primary care physicians | 3 | 8,82 % |
| Radiation oncologists | 6 | 17,65 % |
| Radiologists | 1 | 2,94 % |
| Multiple | 15 | 44,12 % |

**Table 3: Research methodology (N=34)**

|  | **f** | **%** |
| --- | --- | --- |
| Comparative | 6 | 17,65 % |
| Developmental + comparative | 1 | 2,94 % |
| Developmental + evaluative | 12 | 35,29 % |
| Evaluative | 14 | 41,18 % |
| N/A | 1 | 2,94 % |

**Table 4: Effects of TEL on teaching/learning processes (N=34)**

|  | **f [partially]** | **%** |
| --- | --- | --- |
| **Kirkpatrick model of training evaluation** | | |
| level 1 - reaction | 22 | 64,71 % |
| level 2 - learning | 18 | 52,94 % |
| level 3 - behaviour | 4 | 11,76 % |
| level 4 - results | 1 | 2,94 % |
| N/A | 5 | 14,71 % |
| **Results** | | |
| **Level 1** | | |
| The training was satisfactory. | 15 [6] | 44,12 % [17,65 %] |
| The digital tool was useful. | 10 [1] | 29,41 % [2,94 %] |
| Challenges have been reported. | 18 | 52,94 % |
| **Level 2** | | |
| Knowledge has been improved. | 9 [4] | 26,47 % [11,76 %] |
| Skills have been improved. | 5 [1] | 14,71 % [2,94 %] |
| Confidence has increased. | 7 [1] | 20,59 % [2,94 %] |
| Self-efficacy has increased. | 2 | 5,88 % |
| **Level 3** | | |
| The training changed the learners' daily practice. | 4 | 11,76 % |
| **Level 4** | | |
| The targeted recruitment approach was more cost-efficient. | 1 | 2,94 % |

**Figure: Author distribution of the countries**


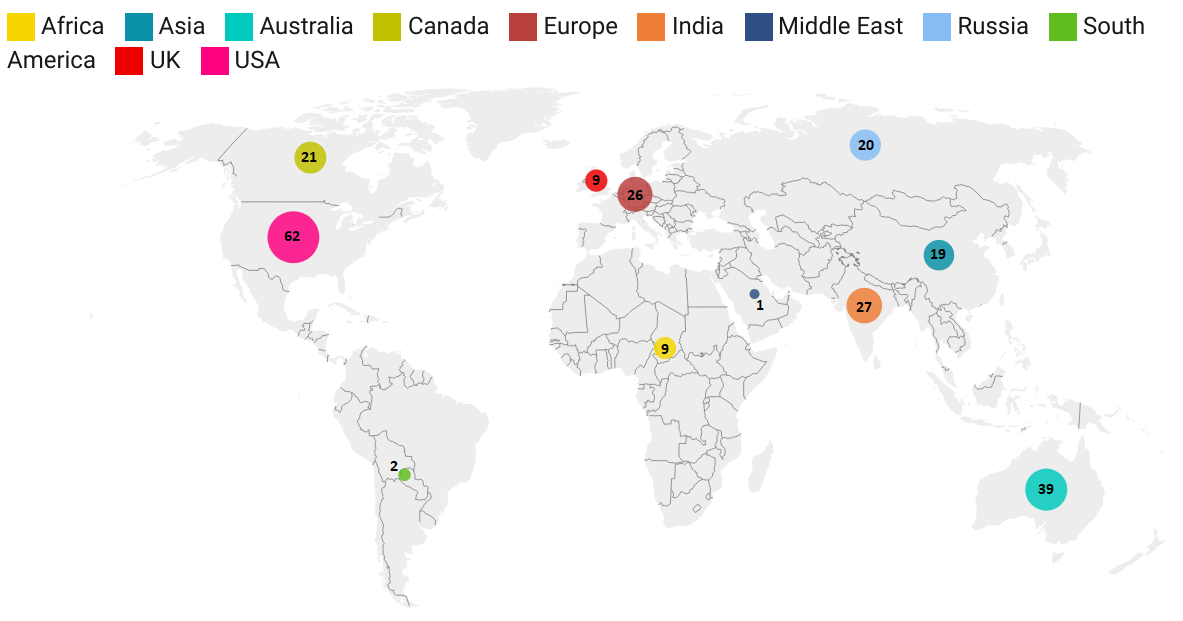

Supplement: Supplementary file 3 — Supplementary file3 (DOCX 132 KB) [file 13187_2023_2329_MOESM3_ESM.docx]
